# North Texas Medical Association

**Published:** 1894-12

**Authors:** 


### North Texas Medical Association.

DALLAS, TEXAS, November 15, 1894.

DEAR DOCTOR:—The next semi-annual meeting of the North Texas Medical Association will be held in Gainesville, beginning at 5 p. m., Tuesday, December 11, 1894.

Encouraged by the great amount of valuable work done at previous meetings, the members anticipate much benefit from the next.

The City of Gainesville is easily accessible by a number of railroads, and is, in many respects, a delightful place for our meeting.

Let every physician, who is desirous of keeping abreast with progressive medicine, attend. Be assured that the work already promised will amply repay any one for the time spent there.

These meetings are the source of valuable facts and information, obtainable in no other way.

All regular, reputable physicians are cordially invited to be present and contribute something in the way of a paper, or report cases.

You will receive a hearty welcome both from the members of this body and the citizens of Gainesville.

R. H. CHILTON, M. D., President, Dallas, Tex.

S. F. KING M. D., Secretary, Sherman, Tex.

### PROGRAMME.

#### SECTION ON THE PRACTICE OF MEDICINE.

"Treatment of Typhoid Fever," by S. D. Moore, M. D., Van Alstyne, Texas.

"Diphtheria," by J. D. Burt, M. D., Farmersville, Texas.

"Pernicious Malaria," by J. O. Mathers, M. D., Howe, Texas.

#### SECTION ON OBSTETRICS AND GYNECOLOGY.

"————————," by T. W. Wiley, M. D., McKinney, Texas.

"————————," by Joe D. Becton, M. D., Plano, Texas.

"Painful Menstruation—Its Cause and Alleviation," by J. W. Carey, M. D., Whitesboro, Texas.

#### SECTION ON SURGERY.

"Supra-Pubic Cystomy and Removal of Prostate Gland," by F. D. Thompson, M. D., Fort Worth, Texas.

"Correcting Deformities of the Knee Joint," by M. M. Edmonson, M. D., Dallas, Texas.

"Enlarged Tonsils in Children and the Importance of Early Removal," by W. M. Moore, M. D., Paris, Texas.

---

### VOLUNTEER PAPERS.

### TO SECTION ON PRACTICE OF MEDICINE.

"Valvular Lesions of the Heart," by J. W. Largent, M. D., McKinney, Texas.

"Disturbance of the Sympathetic Nervous System," by Prof. J. B. Marvin, M. D., Louisville, Ky.

"Treatment of Typhoid Fever," by B. F. Spencer, M. D., Weston, Texas.

"Diphtheria," by W. R. Mathers, M. D., Rock Hill, Texas.

"Pernicious Malaria," by W. L. Higginbotham, M. D., Jamison, Texas.

"A Frequent Mistake in Diagnosis," by Ellen Lawson Dabbs, M. D., Fort Worth, Texas.

"Dyspepsia and its Sequella," by W. R. Howard, M. D., Fort Worth, Texas.

"Delayed Resolution in Pneumonia," by P. F. Ellis, M. D., Bells, Texas.

"Differentiation of the Continued Fevers of Texas," by W. D. Patton, M. D., Bowie, Texas.

### TO SECTION ON SURGERY.

"Antiseptic Treatment of Wounds in Private Practice," by J. E. Gilcreest, M. D., Gainesville, Texas.

"Compound Fracture of Elbow Joint," by J. T. Wilson, M. D., Sherman, Texas.

"Examination of the Eye in the Diagnosis of Intracranial Affections," by John O. McReynolds, M. D., Dallas, Texas.

"Report of Case of Minie Ball Weighing One Ounce—Thirty Years in Antrum of Highmore," by J. R. Floyd, M. D., Paradise, Texas.

---

The officers of every regular medical society are urgently requested to send the undersigned the following: Officers and address of each. Please state if auxiliary to the State Medical Society.

WM. B. ATKINSON, M. D.,
1400 Pine St., Philadelphia, Pa.

